# A novel indicator of selection *in utero*

**DOI:** 10.1093/emph/eoad018

**Published:** 2023-07-19

**Authors:** Ralph Catalano, Tim A Bruckner, Alison Gemmill, Joan A Casey, Claire Margerison, Terry Hartig

**Affiliations:** School of Public Health, University of California, Berkeley, Berkeley, CA, USA; Program in Public Health and Center for Population, Inequality and Policy, University of California, Irvine, Irvine, CA, USA; Johns Hopkins Bloomberg School of Public Health, Baltimore, MD, USA; Environmental Health Sciences, Columbia University Mailman School of Public Health, New York, NY, USA; Epidemiology & Biostatistics, Michigan State University, East Lansing, MI, USA; Institute for Housing and Urban Research, Uppsala University, Uppsala, Sweden

**Keywords:** spontaneous abortion, selection *in utero*, COVID-19

## Abstract

**Background and objectives:**

Selection *in utero* predicts that population stressors raise the standard for how quickly fetuses must grow to avoid spontaneous abortion. Tests of this prediction must use indirect indicators of fetal loss in birth cohorts because vital statistics systems typically register fetal deaths at the 20th week of gestation or later, well after most have occurred. We argue that tests of selection *in utero* would make greater progress if researchers adopted an indicator of selection against slow-growing fetuses that followed from theory, allowed sex-specific tests and used readily available data. We propose such an indicator and assess its validity as a dependent variable by comparing its values among monthly birth cohorts before, and during, the first 10 months of the COVID-19 pandemic in Sweden.

**Methodology:**

We apply Box–Jenkins methods to 50 pre-pandemic birth cohorts (i.e., December 2016 through January 2020) and use the resulting transfer functions to predict counterfactual values in our suggested indicator for selection for ten subsequent birth cohorts beginning in February 2020. We then plot all 60 residual values as well as their 95% detection interval. If birth cohorts in gestation at the onset of the pandemic lost more slow-growing fetuses than expected from history, more than one of the last 10 (i.e. pandemic-exposed) residuals would fall below the detection interval.

**Results:**

Four of the last 10 residuals of our indicator for males and for females fell below the 95% detection interval.

**Conclusions and implications:**

Consistent with selection *in utero*, Swedish birth cohorts in gestation at the outset of the COVID-19 pandemic included fewer than expected infants who grew slowly *in utero*.

## INTRODUCTION

In countries with high quality, universally available prenatal care, at least 10% [1] and as high as 20% [2] of clinically detected pregnancies end spontaneously without a live birth. More than half of spontaneously aborted fetuses appear to be phenotypically indistinguishable from those that survive to birth [3–5]. Relatively slow growth appears their only detectable ‘risk factor’ for spontaneous abortion [6–11]. Literature at the intersection of evolution, medicine and public health includes the argument that the spontaneous abortion of slow growing but otherwise normal fetuses arises, at least in part, from heritable mechanisms conserved because they avert maternal investment in infants unlikely to thrive in prevailing environments [12, 13]. This ‘selection *in utero*’ would target slow-growing fetuses because, if born live, small infants die more frequently than others in the same birth cohorts [7, 9, 14, 15].

Spontaneous abortion reportedly occurs disproportionately among women suffering from stressful events [16, 17]. Its incidence also varies over time with stressors on the population [13]. These circumstances have led to the argument that population stressors raise the standard of how quickly a fetus must grow to warrant continuation of gestation [13, 18]. A small fetus born live in a benign environment might, therefore, have suffered spontaneous abortion during more stressful times.

Tests of selection *in utero* in stressed populations must use indirect indicators of fetal loss in birth cohorts because vital statistics systems typically register spontaneous abortions at the 20th week of gestation or later, well after most have occurred. These bespoke indicators have typically assumed that selection *in utero* requires faster growth of males than of females [14, 18–20]. This assumption is based on an observed excess of small-for-gestational-age males among clinically recognized spontaneous abortions. This excess has led many researchers to use the secondary sex ratio (male divided by female live births) as an indicator of the depth of selection in birth cohorts. The sex ratio, however, may vary owing to changes in either the denominator or the numerator. This circumstance raises uncertainty regarding whether changes in the survival of male or female fetuses account for variation over time in the sex ratio [21].

We assume that scholars interested in how evolutionary mechanisms measurably affect the health of contemporary populations would want more definitive tests of selection *in utero* than currently found in the literature. Such testing would presumably use indicators of the depth of selection in birth cohorts as either a dependent [12] or predictor variable [22]. Here we argue that confirmatory tests of selection *in utero* would make greater progress if the field adopted an indicator of the depth of selection in conception or birth cohorts that (i) faithfully followed from theory, (ii) allowed for sex-specific tests and (iii) used data readily available across many populations. We propose such an indicator below. To encourage and inform consideration of the indicator, we also assess its association with a known population stressor. We compare the indicator’s values among monthly birth cohorts before and during the first 10 months of the COVID-19 pandemic in Sweden—a time when women of reproductive age, like most other Swedes, feared they and close others would encounter a lethal exogenous stressor.

Any indicator of selection against slow-growing fetuses in birth cohorts should reflect the fact that low birthweight varies over time with the incidence of births before 39 complete weeks of gestation. Although these ‘early’ infants weigh less as a group than infants born later in gestation, most also appear at or near the correct size for their gestational age. To ensure that our indicator faithfully reflects the theory of selection against slow-growing fetuses, we adjust the number of low-weight births for the number born preterm and early term. More specifically, we derive our indicator of tolerance for slow growth in historical birth cohorts from the regression of cohort counts of low-weight infants (i.e. birthweight less than 2500 g) on the count of preterm infants (i.e. <37 weeks of gestation) as well as on the count of early-term births (i.e. 37 to <40 weeks of gestation). The fitted value of this regression estimates the number of low-weight infants expected from the size of the cohort and the number of short gestations. Subtracting the fitted from observed values yields residuals from which we then remove ‘autocorrelation’ or patterns in time such as trends, seasonality and the tendency to remain elevated or depressed after high or low values. We reason that negatively signed residuals, which imply unexpectedly few low-weight infants in birth cohorts, occur, at least in part, because selection *in utero* raised the standard of growth needed to continue gestation. If the onset of a lethal pandemic deepened selection *in utero* in Sweden, residuals for cohorts born from February through November 2020 would be significantly negative.

## METHODS

### Data

Using data from the Swedish National Medical Birth Register [23], we constructed monthly, sex-specific time series of counts of low weight (i.e. <2500 g) as well as of preterm (gestation age <37 weeks) and early term (i.e. gestational age 37–39 weeks) live singleton births. Our analyses focused on the 60 months from December 2015 through November 2020. The National Board of Health and Welfare [24] reports that the register includes ‘practically all deliveries’ in Sweden. Reporting requirements have long ensured data of high quality, with little undercounting [25].

### Analyses

We defined ‘exposed’ monthly birth cohorts as those in gestation when Swedish authorities reported the country’s first COVID-19 case (i.e. 31 January 2020). Selection *in utero* predicts that these cohorts would have yielded fewer lower-weight live births than expected from history and from the number of preterm and early-term births in the cohorts. Virtually all gestations end by their 44th week (using the last menstrual period method for estimating gestational age). The first exposed monthly conception cohort would, therefore, have been conceived in April 2019 and all its live-born members would have been delivered by the end of February 2020. The last exposed conception cohort would have been conceived in January 2020 and all its live-born members delivered by the end of November 2020. We did not include Infants delivered after November 2020 in our test because they would have been conceived after the onset of the pandemic and, therefore, could have had heritable risks for fetal death different from cohorts conceived earlier. The difference arises because risk-averse persons of reproductive age would have contributed fewer gestations to post- than to pre-January 2020 conception cohorts.

We applied an interrupted time-series design by proceeding through the following steps (separately for males and females). First, we regressed monthly low-weight (i.e. less than 2500 g) births on monthly preterm (i.e. <37 weeks of gestation) and early-term (37–39 weeks of gestation) births for 50 months (December 2016 through January 2020) prior to the onset of the pandemic in Sweden. Fifty months provide sufficient study power to detect and model autocorrelation.

Second, using the methods of Box and Jenkins [26] we inspected the residuals of the regression for autocorrelation. Epidemiologists use these methods to identify and model temporal patterning in indicators of population health [27]. We applied these methods to our data because prior research during the COVID-19 era in the USA and elsewhere finds that perinatal outcomes exhibit trend, seasonality and the tendency for high or low values to persist into subsequent time periods [28–31]. Failure to control for such patterning would violate the assumption, made by tests of association, of serially independent error terms.

Box and Jenkins [26] offered a general theory of autocorrelation, a common notation for models describing patterns in time-series data, and, most important, rules for determining which model best describes autocorrelation in an observed set of serial measurements. These models express a value observed at time *t* as a function of values observed at time *t* − *n*. Seasonality in a monthly time series, for example, would yield an *n* of 12 in *t* − *n*. Box and Jenkins modeling uses ‘moving average’ and ‘autoregressive’ parameters to gauge how far into the future a high- or low-value influences subsequent observations. Moving average parameters efficiently describe short ‘memory’ of high or low values while autoregressive parameters better fit longer memory.

Third, we estimated equations (i.e. Box–Jenkins ‘transfer functions’) specified by adding moving average and autoregressive parameters, indicated by the results of step 2, to the regression equation estimated in step 1. The residuals of these estimations have a mean of 0, appear normally distributed, and exhibit no autocorrelation.

Fourth, we applied the transfer functions estimated in step 3, with parameters fixed to those fitting the 50 pre-pandemic cohorts, to all 60 cohorts born in the test period (i.e. December 2015 through November 2020).

In step 5, we plotted all 60 residuals of the step 4 estimation as well as their 95% detection interval. If, as selection *in utero* predicts, cohorts in gestation in February 2020 yielded fewer low-weight infants than expected, more than 1 of the last 10 residuals would fall below the lower detection interval.

## RESULTS

**Figure 1.**
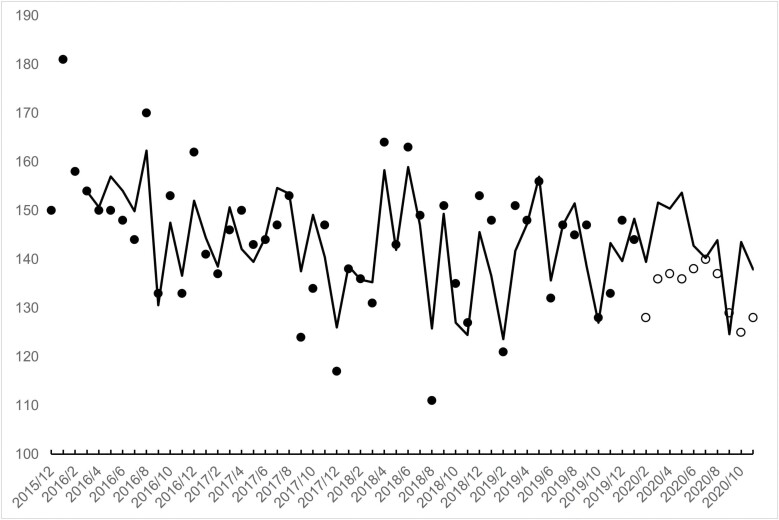
Monthly observed (points) and expected (line) **male** low-weight births in Sweden for 60 months (12/2015 through 11/2020). Cohorts *in utero* at onset of the pandemic (i.e. February 2020) shown as unfilled points

**Figure 2.**
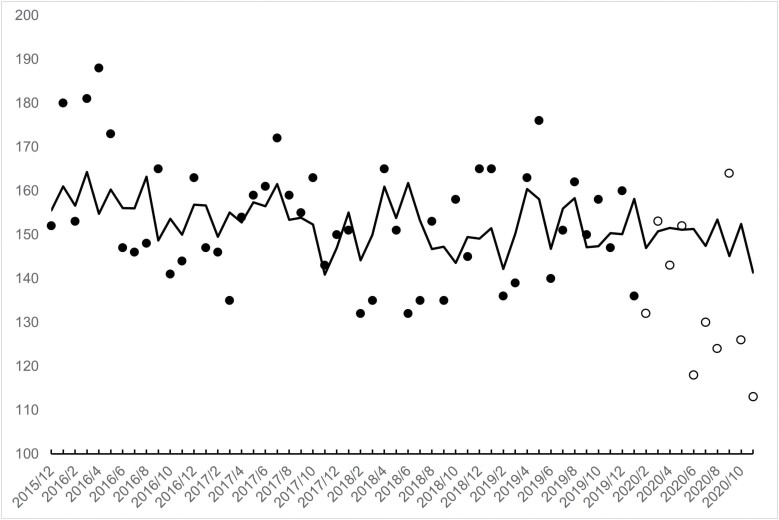
Monthly observed (points) and expected (line) **female** low-weight births in Sweden for 60 months (12/2015 through 11/2020). Cohorts *in utero* at onset of the pandemic (i.e. February 2020) shown as unfilled points

Figures 1 and 2 show, as points, the observed counts of low-weight births for the 60 months in our test period for males and females. Counts for males ranged from 111 to 181 with a mean of 143. For females, counts ranged from 113 to 188 with a mean of 150.

Steps 1–3, in which we identified and estimated Box–Jenkins transfer functions for the 50 months prior to the onset of the COVID-19 pandemic in Sweden, yielded the following transfer functions, in which all estimated parameters exceeded twice their standard errors.

Males: *Y*_mt_ = 32.266 + 0.288*X*_1t_ + 0.018*X*_2t_ + (1 − 0.530*B*)(1 − 520*B*^9^)/(1 − 0.258*B*^3^)*e*_*t*_

*Y*_mt_ is the number of low-weight males born live in month *t*. 32.266 is a constant. *X*_1t_ is the number of preterm males born live in month *t*. *X*_2t_ is the number of early-term males born live in month *t*. *e*_*t*_ is the residual of the transfer function at month *t*. *B*^*n*^ are backshift operators or the value of *e*_*t*_ at months *t* − 1, *t* − 9 and *t* − 3. 0.530 and 0.520 are moving average parameters and 0.258 is an autoregressive parameter.

Females: *Y*_ft_ = 76.895 + 0.211*X*_1t_ + 0.011*X*_2t_ + *e*_*t*_

*Y*_ft_ is the number of low-weight females born live in month *t*. 76.895 is a constant. *X*_1t_ is the number of preterm females born live in month *t*. *X*_2t_ is the number of early-term females born live in month *t*. *e*_*t*_ is the residual of the transfer function at month *t*.

The transfer function for females includes no moving average or autoregressive parameters because we detected no autocorrelation in the residuals of the regression estimated in step 2. The residuals for males, however, showed autocorrelation in which high or low values in month *t* persisted into *t* + 1 and ‘echoed’, although diminished, at *t* + 3 and *t* + 9 months.

Figures 1 and 2 show the results of step 4. The lines in the figures trace monthly counts of low-weight births expected from applying the transfer functions, shown above, with coefficients fixed to those estimated from 50 pre-pandemic months to the entire 60 test months.

**Figure 3.**
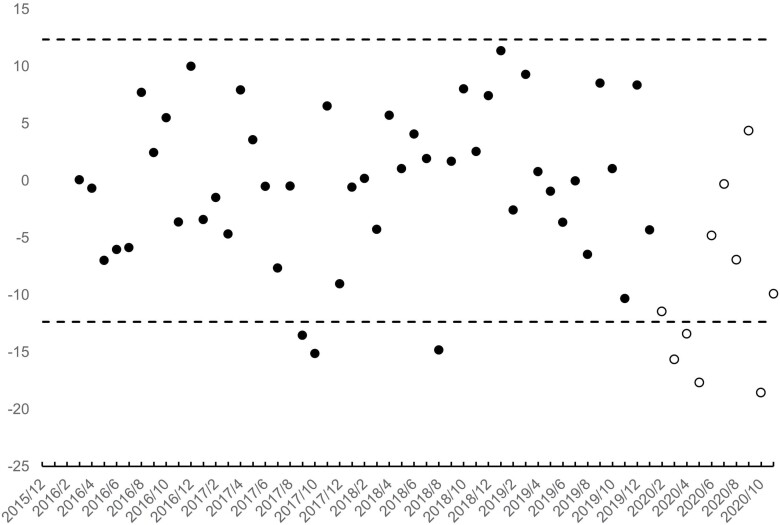
Residual low-weight male births (points) for 60 monthly Swedish birth cohorts (12/2015 through 11/2020). Cohorts *in utero* at onset of the pandemic (i.e. February 2020) shown as unfilled points. 95% detection interval shown with dashed lines

**Figure 4.**
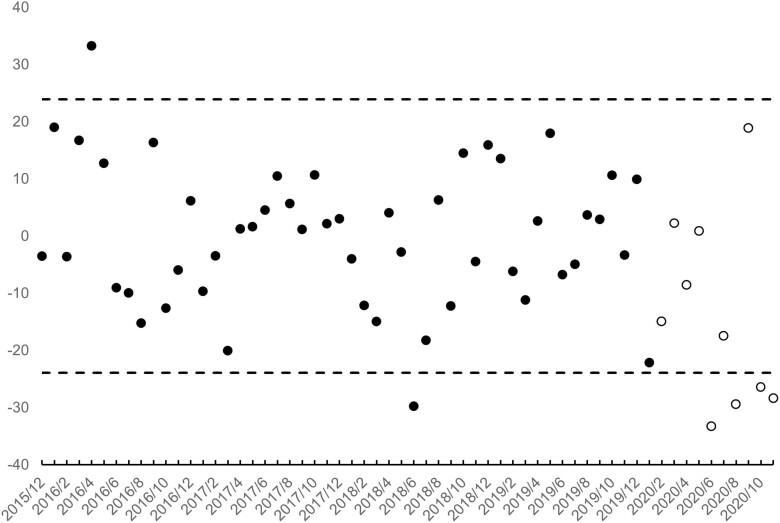
Residual low-weight female births (points) for 60 monthly Swedish birth cohorts (12/2015 through 11/2020). Cohorts *in utero* at onset of the pandemic (i.e. February 2020) shown as unfilled points. 95% detection interval shown with dashed lines

Figures 3 and 4 show the residuals of the step 4 estimation as well as their 95% detection interval. Residuals for birth cohorts in gestation during February 2020 show as unfilled points. Consistent with selection *in utero*, 4 of the 10 male (i.e. those born in March, April, May and October 2020) and four of the female (i.e. those born in June, August, October and November 2020) birth cohorts in gestation at the onset of the pandemic yielded fewer low-weight births than expected. ‘Missing’ low-weight male births for the 4 cohorts summed to 65, or about 11% of the 600 expected in those cohorts. The 118 missing low-weight females equalled about 20% of the 598 expected.

## DISCUSSION

Our cohort indicator of the depth of selection *in utero* follows faithfully from the theory describing selection *in utero* [13, 14, 18, 21, 22, 28], can describe males or females and can be constructed from widely available data. We also show here that the indicator fell, as theory predicts, when the onset of the COVID-19 pandemic stressed Swedes.

We acknowledge that the accuracy of birth certificate reports of gestational age, particularly before 36 weeks of pregnancy, may vary over time and across places. We note, however, that we attempt to compensate for this problem by suggesting an indicator inferred primarily from birth weight, which appears more dependably measured than gestational age. Our indicator uses gestational age only to identify births before 40 weeks gestation—a relatively less controversial, although likely imperfect, determination.

We do not offer our findings as strong evidence that selection *in utero* increases spontaneous abortion during stressful times. Our observational test cannot rule out that some non-adaptive mechanism induced the association we found. We note, for example, that our finding of less ‘tolerant’ male and female birth cohorts in October 2020 could arise if the onset of the COVID-19 pandemic somehow impeded reproduction among individuals with traits that increase the likelihood of fetal growth retardation. Under this circumstance, conceptions in February 2020 would be ‘scheduled’ for a term birth in October 2020. We expect that the rapidly expanding literature on effects of the pandemic on reproductive choices will shed light on the characteristics of the October 2020, and subsequent, birth cohorts in Sweden and elsewhere.

Unlike its neighboring countries, Sweden imposed relatively few COVID-19-related restrictions. Gyms, schools, restaurants and shops all remained open throughout the pandemic [32]. This strategy, which relied on voluntary compliance with recommendations (as opposed to requirements), could have induced a stress response among pregnant women that differed from that in other high-income societies. Indeed, the Swedish approach aimed to reduce the anticipated deleterious effects of a prolonged lockdown on mental health and relationships. We, therefore, caution against generalizing our results to other countries.

Although fetuses of both sexes spontaneously abort throughout gestation, early losses appear dominated by females and later losses by males [33]. The onset of the pandemic would induce detectable loss among female fetuses earlier in gestation than among male fetuses. Consistent with this prediction, we found that male *birth* cohorts with fewer than expected low-weight births appeared near in time to the onset of the pandemic (i.e. March, April, May and October 2020) while female cohorts with fewer than expected low-weight births generally appeared later (i.e. June, August, October and November 2020). Further testing of this patterning will require difficult-to-obtain 2020 data describing the sex and timing of pregnancy losses before 20 weeks. Clinical registers in Scandinavia may provide an opportunity to pursue this important avenue of research [34].

Strengths of our analysis include the use of a large population with consistent and high-quality data collection protocols. Our methods also control for well-documented autocorrelation in birth outcomes that could induce spurious results.

We offer our indicator as an aid for testing the prediction of greater selection in acutely stressed populations. Theory regarding selection *in utero* [13] does not, however, confine itself to acute stressors. It assumes that females spontaneously abort when a live birth would otherwise produce an infant unlikely to thrive in the prevailing environment. This implies that chronic stressors encountered by pregnant females will also affect the survival of low-weight fetuses. We also note that chronic stressors likely condition a population’s response to an acute stressor. The onset of, as in our test, a lethal pandemic could, therefore, have different effects on populations varying in exposure to persistent stressors such as poverty, extreme climate conditions or sectarian strife. Although our indicator could serve as an outcome in tests of the main and interaction effects of acute and chronic stressors, we anticipate that specifying exposures in such tests will prove challenging.

The next logical step in this line of research would test whether our indicator correlates with, as theory predicts, cohort infant morbidity and mortality. We note, however, that COVID-19 cohorts may not be appropriate for such a test given the possibility that their members may have suffered unusually high rates of infectious illness, and its related morbidity, in infancy. A test of whether selection *in utero* produces ‘culled’ cohorts with relatively improved infant health and survival would better consider cohorts stressed by non-infectious shocks [22].
